# Supplementary material for: All-at-once RNA folding with 3D motif prediction framed by evolutionary information
Source: Nat Methods. 2025 Oct 3;22(10):2094–106. doi: 10.1038/s41592-025-02833-w (PMC12510878; doi:10.1038/s41592-025-02833-w)
Supplement: Supplementary file 3 — Source code to run CaCoFold-R3D. Data and results. [file 41592_2025_2833_MOESM3_ESM.gz › supplemental_material/results/CaCoFold-R3D/CaCoFold-R3D_Rfam_vshuffle/RF01983_Pinc.R2R.sto.pdf]

RF01983_Pinc

5′ - C U U U Y C U G A G C ● G G A A A G A G A Y C A G G U ● A A G U A G Y A U A U R A U A U C C U U C U U A U U C C C A Y G C A G A U G U C U G A G G C U U C U Y U Y Y C C U U U G G U C A A A U C U G A U U U G U A U U U C U U C U U G G U U A A U A C C C C C A G A G U G C U C Y A G G C U G C U U U C U G C C U R
